# An analysis of the impact of newborn survival policies in Pakistan using a policy triangle framework

**DOI:** 10.1186/s12961-021-00735-9

**Published:** 2021-05-25

**Authors:** Jamil Ahmed, Carmen Huckel Schneider, Ashraful Alam, Camille Raynes-Greenow

**Affiliations:** 1 Department of Family and Community Medicine, College of Medicine and Medical Sciences, Arabian Gulf University, Manama, Bahrain; 2 Sydney School of Public Health, Faculty of Medicine and Health, The University of Sydney, Sydney, NSW 2006 Australia

**Keywords:** Pakistan, Neonatal health, Newborn survival, Health policies, Health policy analysis, Implementation

## Abstract

**Introduction:**

Pakistan has made slow progress towards reducing the newborn mortality burden; as a result, it has the highest burden of newborn mortality worldwide. This article presents an analysis of the current policies, plans, and strategies aimed at reducing the burden of newborn death in Pakistan for the purpose of identifying current policy gaps and contextual barriers towards proposing policy solutions for improved newborn health.

**Methods:**

We begin with a content analysis of federal-level policies that address newborn mortality within the context of health system decentralization over the last 20 years. This is then followed by a case study analysis of policy and programme responses in a predominantly rural province of Pakistan, again within the context of broader health system decentralization. Finally, we review successful policies in comparable countries to identify feasible and effective policy choices that hold promise for implementation in Pakistan, considering the policy constraints we have identified.

**Results:**

The major health policies aimed at reduction of newborn mortality, following Pakistan’s endorsement of global newborn survival goals and targets, lacked time-bound targets. We found confusion around roles and responsibilities of institutions in the implementation process and accountability for the outcomes, which was exacerbated by an incomplete decentralization of healthcare policy-making and health service delivery, particularly for women around birth, and newborns. Such wide gaps in the areas of target-setting, implementation mechanism, and evaluation could be because the policy-making largely ignored international commitments and lessons of successful policy-making in comparable regional counties.

**Conclusions:**

Inclusion of clear goals and targets in newborn survival policies and plans, completion of the decentralization process of maternal and child healthcare service delivery, and policy-making and implementation by translating complex evidence and using regional but locally applicable case studies will be essential to any effective policy-making on newborn survival in Pakistan.

## Key messages

- Pakistan’s high rates of newborn mortality could be associated with a lack of clear, inclusive, and objective policy-making processes, and a failure to formulate a comprehensive and clear policy.
- An evaluation of previous and existent policies relevant to newborn survival in Pakistan uncovers several weaknesses reflected in poor implementation of these policies.
- Any future health policy-making with a focus on improving newborn survival in Pakistan or other similar situations may benefit from a translation of evidence, clear target-setting, and decentralization of child healthcare services.

## Introduction

### High burden of newborn deaths in Pakistan

Although child survival has continued to improve globally since 1990, 5.3 million children under the age of five still die every year, and 2.6 million of them die in their first month. Approximately 80% of newborn deaths can be prevented through known effective interventions [1]. Deaths in newborns account for half of all deaths in children under five in 11 countries with a heavy burden, including Pakistan as well as India, Bangladesh, Indonesia, Nigeria, China, the Democratic Republic of the Congo, Ethiopia, Angola, and Kenya. In this group, about 73% of deaths in newborns occur within the first week, about 36% on day 1, and 32% within 6 hours after birth [2]. In these countries, most newborn deaths are due to preterm birth, complications during labour, and infections [3].

**Fig. 1:**
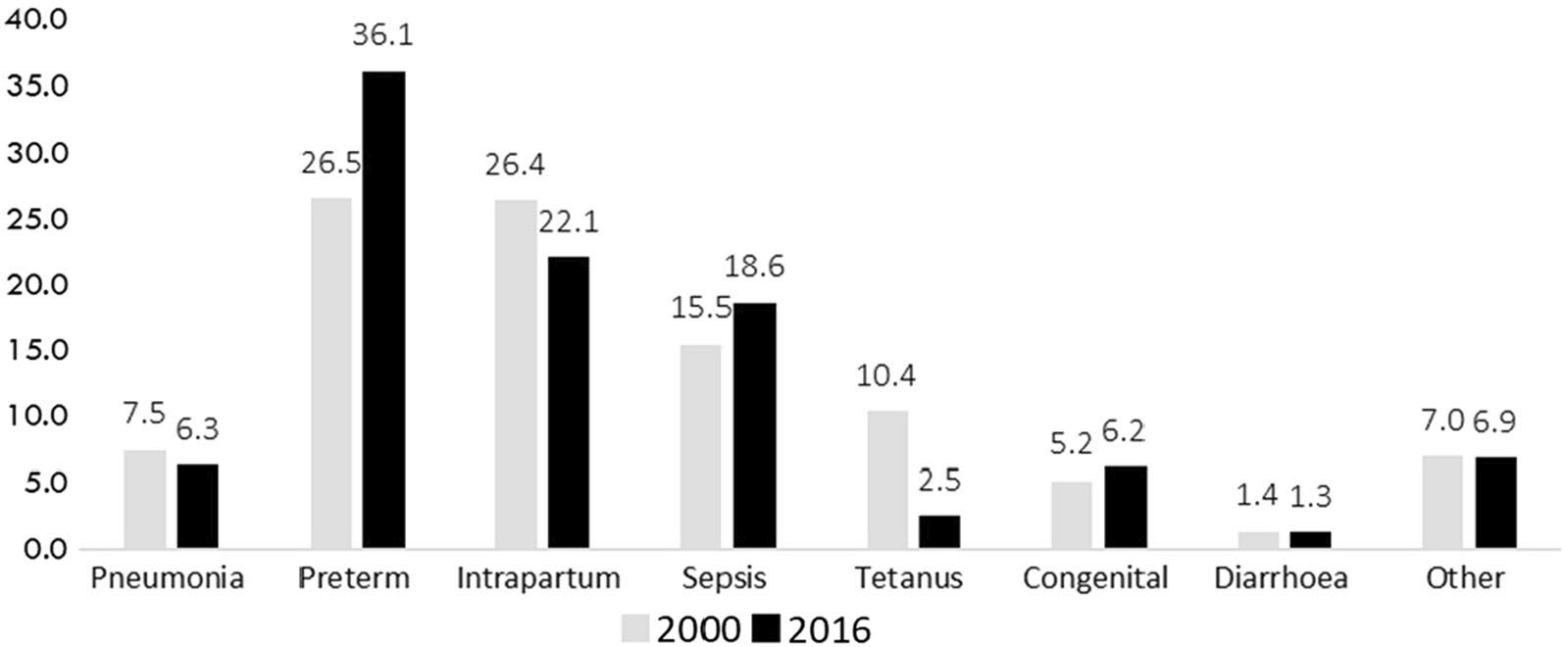
Causes of deaths of newborns in Pakistan (percent) (Source: Estimates generated by WHO and the Maternal and Child Epidemiology Estimation Group, 2017)

The causes of newborn deaths in these high-burden countries has not significantly changed during the previous two decades (Fig. 1), and disparities exist both between and within countries [4]. The risk of dying in the first day of life is higher in rural areas of Pakistan compared to urban areas, and newborns from the poorest households are more than twice as likely to die compared to the richest [1]. The recent Pakistan Demographic and Health Survey found that 62.6% of births in rural areas were conducted by skilled birth attendants compared to 83.8% in urban areas [5]. Although focussed antenatal care is currently being implemented in primary healthcare facilities of some parts of the country, and 86.2% of women now seek advice from a skilled provider during pregnancy [6], access to high-quality essential care around pregnancy and birth continues to be a challenge for the majority of rural populations in the country [7].

### Health system and reform: the policy context

Almost two thirds of Pakistanis utilize private out-of-pocket healthcare services [8]. Pakistan spends 38 US dollars per capita on health annually, against at least 44 US dollars proposed by WHO to ensure peoples’ access to essential health services [9]. The country’s total governmental health expenditure as a proportion of total health expenditure has decreased over the last two decades from 35% to 27.5% [10]. Despite this low governmental spending on health, Pakistan’s reproductive, maternal, newborn, and child health programme budget increased by 181% between the years 2000 and 2010, mostly from foreign donor agencies, such as the United States Agency for International Development and the Department for International Development of the United Kingdom (DFID-UK) [11]. Policy to address newborn mortality has been shaped by this public–private-donor funding mix as well as a broader set of health system reforms over the past two decades [12].

Prior to the major health system reform in 2010–2011, known as the 18th constitutional amendment, leading to decentralization of the functions of health to the provinces, financing, and policy-making in health (and other sectors including education, environment, and rural development) were mainly functions of the federal government. The federal Ministry of Health had a wide range of functions in its mandate which included regulation of healthcare, administration of institutions such as specialized hospitals in the provinces [13], and service delivery through national vertical programmes. Following the constitutional amendment, provinces took on the responsibility of generating and expending their own revenue. In addition to this fiscal autonomy, provinces were transferred functions of health planning and legislation, regulation of the health sector, service delivery, and management of human resources for health [14]. The aim of the reform was to effectively redistribute resources and improve context-specific policy-making, planning, and innovation [14].

The years immediately following the constitutional amendment saw the achievement of some key health financing and planning outcomes as they moved from a federal to provincial level, including the development of health sector strategies. However, issues such as health governance, service delivery, and regulation lagged [14]. The transfer of power to the provincial bodies and the expected change in performance has been sluggish, and decentralization has only been partially implemented. The Primary Care Systems Profiles and Performance (PRIMASYS) case study on Pakistan [14] found that federal vertical programmes on maternal, newborn, and child health (MNCH), immunization, malaria, HIV/AIDS, tuberculosis, blindness, avian flu, hepatitis, and nutrition have yet to be integrated within routine healthcare services, even though their financing mechanisms and administrative affairs have already been devolved to the provinces.

A recent example from the Sindh province highlights the policy uncertainty following partial decentralization and, later, recentralization. This happened in 2019, when the federal government decided to resume administrative control of three major specialized hospitals in Sindh; however, the provincial government disputed the move, citing its own allocation of spending and operational strategy [15]. Due to confusion over both financial and operational responsibility, not only have there been challenges to coordinate the national policy implementation by the provinces after the decentralization, international development partners have also found it difficult to support the efforts by the provinces to improve MNCH [16]. This combination of partial decentralization, disputed governance, and policy uncertainty forms part of the challenge to create feasible and effective policy to address newborn mortality in Pakistan [17]. Therefore, an in-depth analysis of the gaps within the previous health-related policies is crucial to understanding the reasons that could have driven the failure to effectively implement newborn survival strategies in Pakistan. We carried out an analysis of the content of Pakistan’s major policies and strategies during the past two decades, from 2001 to 2019, impacting upon prevention of newborn mortality.

### Methods and analytical approach

We first identified, described, and categorized the existing and past policies that are designed to oversee prevention of newborn mortality, then further analysed and explained the reasons for their impact—or lack thereof—on mortality rates in newborns. We first collated and reviewed the content of all federal-level health policies from 2001 to 2019 for specific policy content relevant to newborn mortality and survival. We then traced how these policies would need to be translated into programmes, services, and interventions for successful implementation. We traced over time the evolution of policy content and the extent to which the changing policy context described above influenced this implementation.

We then further applied the policy content analysis to a case study of health policy and strategy documents of Sindh province. Sindh province was chosen as a suitable case study because the province is typical with respect to health policies, programmes, and healthcare service delivery in Pakistan. An assessment of the provincial newborn mortality prevention strategies and implementation policies was considered useful for understanding how decentralization reforms impacted on policy formation and implementation.

In Sindh province, due to a lack of access to quality healthcare services, child health indicators are worse than the national average. Rural areas of the province particularly face challenges in access to essential maternal and child healthcare. For instance, only 34.2% of women in rural Sindh had completed four or more antenatal care visits, and only 58.2% delivered at a health facility in the 5 years preceding the 2017 Demographic and Health Survey [5].

Lastly, we also conducted an international comparative analysis reviewing the newborn mortality and survival policies in other countries in the region which have achieved consistent success in reducing newborn mortality despite the challenges. We selected India and Bangladesh, which share similar contextual factors, challenges, and health system structures with Pakistan. The health system in these countries also comprises a three-tier system: primary, secondary, and tertiary healthcare system. These three countries have similarities in cultural and sociodemographic context as they were unified before partition of India in 1947 and separation of Bangladesh from Pakistan in 1971. These countries also have experienced a heavy newborn mortality burden in the past; however, they have progressed to significantly reduce it over the past decade, although they continue to have a newborn mortality rate which is higher than most of the high-income countries. We did this by searching for the most recent literature on policies guiding newborn mortality prevention interventions on the websites of ministries of health and United Nations agencies of these countries. A hand search was conducted of the relevant health policy journals and websites of the nongovernmental organizations and health ministries of these countries by using specific key words like “newborn” or “neonatal” and “survival” or “mortality” and “policies” and “India” or “Bangladesh”. We specifically sought instances of policy and system antecedents that enabled the implementation of interventions and examined how these could be developed in Pakistan.

### Data collection

**Fig. 2:**
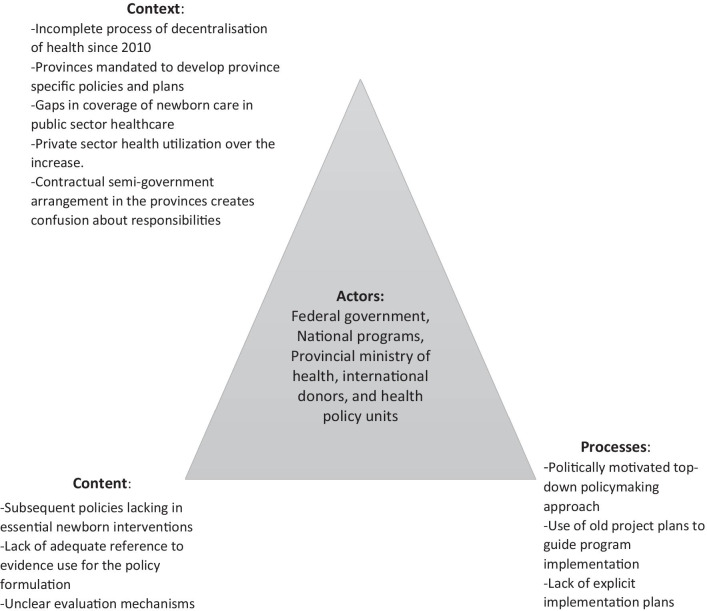
Policy triangle framework describing the actors, content, context, and processes within Pakistan’s newborn mortality prevention policy policies, strategies, and implementation

We included both general health strategies, policies, and planning documents as well as any specific MNCH policies in our analysis, which was carried out during November 2019 and April 2020. Documents developed during the period from 2001 to 2019, both before and after the decentralization of healthcare, were included. These documents were subsequently categorized as policy plans, health policies, and policy strategies. We mainly relied on online resources for the availability of the targeted health policy documents and publications and searched for documents on the websites of the Ministry of Health (prior to decentralization), national programmes on MNCH, and the National Lady Health Workers and Family Planning Programme, as well as nongovernmental organizations (for project evaluation reports, for instance). We then searched reference lists and snowballed further policy documents. We specifically reviewed the content of these documents for the provision of newborn mortality prevention policies, plans, strategies, newborn health service delivery-related policies, and blueprints containing allocation of resources and indication of responsibilities for the implementation of interventions. We also looked for the policy and programme documents from international development agencies such as the United Nations International Children's Emergency Fund (UNICEF), WHO, and other nongovernmental organizations with an influence upon maternal and child health in Pakistan. We included seven federal government documents, two Sindh provincial policy and strategy documents, and one international action plan and a progress report in our analysis. For some unanswered questions and unclear items related to the MNCH Planning Commission 1 (PC-1) and its subsequent implementation, the first author contacted an official in the MNCH programme of Sindh province who was believed to give detailed insight into the current situation of the MNCH programme and another official in a United Nations agency which supports the development of provincial newborn survival strategies in Pakistan. The officials were identified through personal contacts and contacted by phone, and notes were taken which were incorporated into the analysis [18] (Box 1). A policy triangle approach originally prescribed by Walt and Gibson in 1994 [19] (Fig. 2) was used to analyse the context, content, processes, and actors which influenced these policies. Context could mean national, provincial, or even international political, economic, social, and cultural factors which may affect a health policy. Content is the matter in the evaluated policy including what areas of healthcare the policy addresses and what areas it does not. Process defines the mechanism by which these policies were formulated, aimed to be implemented, or evaluated. Actors can be the individuals, communities, groups, institutions, or the government which influences the health policy.

Box 1. Timeline of major health policies and plans evaluated for relevance to newborn survival in Pakistan

1. National Health Policy Pakistan—2001
2. Pakistan National MNCH Programme Planning Commission (PC)-1—2005
3. Sindh Health Policy—2005
4. The Medium-Term Development Framework 2005–2010
5. *Draft* National Health Policy Pakistan—2009/2010
6. 18th Amendment—decentralization of heath functions to provinces 2010–2011
7. Sindh health sector strategy 2012–2020
8. Every Newborn Action Plan 2014
9. Every Newborn Action Plan Progress report (WHO/UNICEF)—2015
10. National Health Vision Pakistan 2016–2025, Pakistan—2016
11. National Vision 2016–2025 for the Coordinated Priority Actions to Address Challenges of Reproductive, Maternal, Newborn, Child, Adolescent Health and Nutrition—2016

## Results

### Newborn mortality prevention within the federal health policies, plans, and strategies in Pakistan

#### National Health Policy 2001

*Context:* Before 2000, health policies in Pakistan had little focus on newborn survival as we could only find most significant policies afterwards. The first main health policy was Pakistan’s National Health Policy 2001 developed by the then federal Ministry of Health [20].

*Content*: The policy listed, under 10 key strategies, a target of reducing the proportion of low-birth-weight babies, a risk factor of newborn mortality, from 25 to 15% by 2010. Currently, the national prevalence of babies with low birth weight is 22% which shows that the target is far from achieved [5]. However, despite the rate of infant mortality rate being very high, this policy only mentions the terms “neonatal” or “newborn” once, on page 3, that being with reference to immunization against neonatal tetanus [20].

*Process*: This policy lists, under the broad areas of reforms, implementation approaches for focussed reproductive health services, addressing vaccine-preventable diseases (mainly poliomyelitis), improving service delivery and maternal and child nutrition; however, the policy has no specific item on newborn mortality prevention.

*Stakeholders/Actors*: The policy relied upon provinces for the implementation of targets like recruitment of 100 000 family health workers and upgrading the health facilities by the provinces. The ambitious goals to regulate the private sector, create awareness among masses about public health issues, and build capacity of stakeholders to monitor the implementation of policies have yet to materialize.

### MNCH PC-1

*Context*: A major policy change took place in 2005 when newborn survival was formally integrated into the MNCH programme. The programme was run by the federal government and supported by donor agencies, mainly the DFID-UK [21]. The programme aims were designed to align with the Millennium Development Goals 4 (reduce child mortality by two thirds by 2015) and 5 (improve maternal health by reducing the maternal mortality ratio by 75% by 2015) [22]. The programme’s aim was to enhance poorer communities’ access to quality MNCH services by setting up programmes in the provinces and districts. At its inception, the MNCH programme was envisaged to strengthen and upgrade 650 health facilities to provide basic emergency obstetric and newborn care (BEmONC), and 214 hospitals to provide comprehensive emergency obstetric and newborn care (CEmONC) services [22]. BEmONC services include seven signal functions of administration of parenteral antibiotics, uterotonic drugs, and anticonvulsant drugs for preeclampsia and eclampsia, manual removal of placenta, removal of retained products of conception, performing assisted vaginal delivery, and basic neonatal resuscitation, whereas CEmONC has two additional services of caesarean section and blood transfusion.

*Content*: The programme proposal (also known as PC-1, referred to as the MNCH programme document here) described activities for the programme implementation. The original 2006 MNCH programme document still guides programme implementation today. A budgetary allocation was also approved in 2006 under the MNCH Strategic Framework [23]. The MNCH programme planning document contains some strategies to address newborn survival; however, it acts as a guideline rather than a policy directive.

*Process*: The implementation process listed adding newborn care units to the existing BEmONC and CEmONC facilities. Rural health centres that serve populations of up to 100 000 people and taluka headquarters, which serve 50 000 to 1 million people, were to establish mostly BEmONC services. Tertiary care hospitals in the districts and teaching hospitals, which are usually affiliated with a medical teaching institution, were to have CEmONC services. While details like costs of renovation and construction work, allocation of the equipment and supplies for the newborn care, and staff salaries were listed, the document gives no clear mechanisms for the delegation of responsibilities, accountability, and monitoring and evaluation of the programme activities and objectives. A qualitative study based on interviews with managers of the MNCH programme showed that this poor programme implementation could have arisen due to resource constraints, lack of stakeholder involvement and integration with routine healthcare services, and insufficient devolution of healthcare to lower levels [24].

*Stakeholders/actors*: The MNCH programme document also proposed the deployment of a paediatrician or a neonatologist and a medical officer, two nurses, and other support staff to provide services in the newborn care units to be established in the CEmONC centres. However, since the release of the MNCH programme document in 2006, the establishment and upgrading of the health facilities as well as deployment of the necessary staff to improve and provide newborn care services have been inconsistent, and noticeably lacking progress in rural areas. The donors of this programme commissioned a midterm evaluation of the programme during 2011–2012, which showed that at that point, there was still a need for the establishment of 760 BEmONC facilities, representing a deficit even higher than the initial target. However, this evaluation indicated an achievement towards establishment of a significant number of CEmONC facilities, even though 123 more CEmONC facilities were still required during 2011–2012 [25]. This evaluation also showed that though some upgrades had taken place at tertiary care facilities, the lower, subdistrict-level health facilities had been ignored. This evaluation also concluded that the programme did not fulfil its commitment to reduce child mortality [25].

### National Health Policy 2009

*Context*: In 2009, a second major health policy with relevance to addressing newborn mortality was developed as a draft. The draft National Health Policy 2009 was broad and aimed to create a policy platform for strengthening provincial health strategies in the areas of essential care packages, workforce, health information systems, and health technology and health system governance. This policy too has a clear mention of Millennium Development Goals as the major driving force.

*Content*: The National Health Policy 2009 contained no specific details on commitments of funding, time frames, or support processes with a direct relevance to newborn survival. By 2010, the draft had not been approved because of the ongoing devolution process. Two years after the release of the draft National Health Policy 2009, the Ministry of Health was abolished because of decentralization.

*Process*: Lacking in clearly showing the time frames and implementation strategies, the formulators of the policy anchored the responsibility on a vague cooperation between provinces and the federal Ministry of Health. Newborn mortality prevention strategy implementation was not identified, except that the policy recognizes that the EmONC will be a priority area for health facilities.

*Stakeholders/actors*: Like most other federally developed policies, this policy also followed the tradition of saying that the “Federal Health Ministry will support the provincial and district governments in monitoring its implementation for effectiveness”.

### Every Newborn Action Plan (WHO/UNICEF)

*Context*: By 2014, no further newborn survival policies had been developed on the federal level. The WHO/UNICEF Newborn Action Plan was an important international-level document that was used as the key guiding policy for newborn survival [26]. This action plan set two primary goals (ending preventable newborn deaths and ending preventable stillbirths) as well as five strategic objectives for achieving those goals (Box 2).

*Content:* As a first step in progressing the action plan, a review of the current state of affairs in the highest-burden countries was undertaken and published in 2015. That report, the Every Newborn Progress Report 2015 [27], found that, at the time, Pakistan still did not have a national policy to address a high newborn mortality rate (although it was reported as in progress). However, some relevant national-level policies had progressed. These included adding newborn lifesaving commodities in the National Essential Medicines List and Logistics Management Information Systems.

*Process:* The report also highlighted the implementation process envisaged for this action plan. Under this plan, Pakistan was planning capacity-building activities, and research and strengthening of special newborn care units, although the time frames for these interventions were not specified.

*Stakeholders/actors*: The action plan was endorsed by 194 countries in 2014, including Pakistan. As part of its commitment to the Newborn Action Plan, Pakistan became one of the major stakeholders, and one of the 51 countries that started the biannual completion of the Every Newborn Action Plan Country Progress Tracking Tool.

Box 2. Every Newborn Action Plan strategic objective (endorsed by the 194 WHO Member States, including Pakistan, in 2014)

1. Strengthen and invest in maternal and newborn care during labour, birth, and the first day and first week of life.
2. Improve the quality of maternal and newborn care.
3. Reach every woman and newborn to reduce inequities.
4. Harness the power of parents, families, and communities.
5. Count every newborn through measurement, programme tracking, and accountability.

### National Health Vision Pakistan 2016–2025

*Context*: This is the broad federal-level health policy currently in the country [28]. Developed 15 years after the earlier National Health Policy in 2001, this document acknowledged the challenges and lack of progress following decentralization.

*Content*: National Health Vision Pakistan, under the Every Newborn impact framework, identifies the care around birth and care of small and sick newborns as important outcomes impacting newborn survival. The vision is clearly inspired from the global every newborn action plan but gives little guidance on how to implement the vision or if, and how, federal government would support the provinces. The document does not contain any service-level, procedural, or intermittent targets to monitor progress.

*Process*: Overall, the development of the national vision was driven by a sense of limited control over healthcare systems in the provinces after the decentralization and a realization that country’s health indicators were stagnant.

*Stakeholders*: The national vision document does not address how the private sector, a major provider of health services in Pakistan, and other stakeholders beyond healthcare would be involved in achieving the 10 highly generic, priority actions it lays out. Although the vision document stipulated that health service delivery would remain a provincial obligation, it also cited the re-establishment of a health ministry as the Ministry of National Health Services, Regulation and Coordination.

### Miscellaneous health-related policies with a possible impact on newborn health

*Context*: In addition to the policies reviewed above, we also identified several other policies that also have relevance to newborn survival. These included the National Reproductive Health Services Packages policy 2000, Protection of Breastfeeding and Young Child Nutrition Ordinance, 2002, the Punjab Reproductive, Maternal, Neonatal and Child Health Authority Act, 2014 (updated 10/04/2014), the Punjab Maternity Benefit Ordinance, the Reproductive Healthcare and Rights Bill 2009, the Khyber Pakhtunkhwa Protection of Breastfeeding bill, 1958 (updated 28/02/2012), and Child Nutrition Act in 2015. However, we were unable to locate more recent national-level policies that specifically address newborn mortality.

*Content*: Most of the content of these policies had an indirect reference to child health mortality prevention with a little emphasis upon specific newborn mortality prevention interventions. A review of the *Medium-Term Development Framework for 2005 and 2010* [29] also contains a generic reference to the government’s commitment to curtailing child mortality; however, a specific newborn survival item could not be found under its broad agenda.

*Process*: Despite the ambitious aims of these policies and bills, they lacked specific details about what processes would ensure their implementation and evaluation in the provinces.

*Stakeholders*: Separate newborn survival strategies based upon the Every Newborn Action Plan, formed with the support of development partners, are currently under various stages of development in the provinces. At least one of the provinces has already submitted its plan for provincial-level budget approval.

A mixed pattern of coverage and implementation of newborn policies was observed in our analysis of these policies. Most of these policies were more generic about child mortality prevention, and specific strategies for newborn survival were missing. The national MNCH programme showcases achievements in upgrades of health facilities and training the midwives up until 2012 [23]; however, the impact of these achievements on newborn mortality has not been realized as shown by poor progress on the newborn mortality indicators. In fact, the maternal and child mortality, particularly newborn mortality, has remained very high for the country over the period from 2001 to date, indicating that these achievements may not have been sustained, that policies did not transform into effective implementation plans, and that population access to quality services during pregnancy and birth was probably limited. During these years, maternal and child health indicators have worsened, and current evidence even shows that the routine health information system grossly underestimates the actual burden of the maternal and child mortality rates. For example, a study that employed an enhanced surveillance system to count the actual number of neonatal deaths in a rural district of Pakistan calculated a neonatal mortality rate of 40 compared to the routinely reported 20 per 1000 live births in the district during the study period [30].

After decentralization in 2010, the scope of activities to be included in MNCH was reduced, and provinces were required to develop their own programme documents aligned with this reduced scope. According to one provincial MNCH programme official, the programme functions are now limited to the training of community midwives in midwifery schools of the districts and refresher training of some staff through support from organizations like UNICEF [18].

### Provincial newborn survival strategies: the case of Sindh province

Here, the case of Sindh province is presented considering the question of how strategic and programmatic documents from the province address the delivery of newborn healthcare services, and how implementation at the provincial level has been affected by the broader policy environment. Sindh province is predominantly a rural population relying on the rural healthcare system for providing maternal and child health services. Most of the basic health units and some rural health units are currently managed by Peoples Primary Healthcare, a company that came into being as a public–private partnership venture in Sindh in 2007, whereas the secondary level of care continues to be operated by the department of health, creating service delivery working in parallel, with little integration between the two.

### Provincial MNCH programme after the decentralization

*Context*: According to an official in Sindh, the programme faces challenges in remaining committed to the original aims of improving the maternal and child healthcare services after the decentralization, resulting mainly from the loss of financing from its parent federal MNCH programme (the provincial programme received about half of its funding from DFID-UK and half from the federal government before decentralization).

*Content*: As a result of the removal of financial support, the programme has mostly been providing community midwifery education through its 15 district-based midwifery schools and appointing midwives in their respective communities (3500 so far) by supporting them with initial equipment and material to run their stations in rural areas [18].

*Process*: As envisaged in its parent federal programme, the programme’s original aim was to facilitate implementation of maternal and child health interventions across the country. In response to a question about that aim, one of the officials of the Sindh MNCH programme claimed that the programme, by providing human resources and equipment, supported 12 district and 31 taluka (subdistrict) headquarters hospitals to enhance CEmONC and 15 subdistrict hospitals and 121 rural health centres to function as BEmONC service-providing facilities.

*Stakeholders/actors*: The presence of multiple stakeholders in the public health sector has given rise to a state of confusion around a global responsibility to improve newborn health. Our personal communication with stakeholders such as those in the Sindh MNCH programme showed that these stakeholders were not clear about which districts were delivering BEmONC and CEmONC services that were established by the programme in the districts [18].

### The Sindh Health Policy 2005

*Context*: This policy also recognized that child health was linked with the implementation of the Millennium Development Goals, a maternal and child healthcare package consisting of training community midwives and making pregnancy safer.

*Content*: The Sindh Health Policy 2005 [31] consisted of 22 major priority areas. While newborn survival was not specifically included, several items including control of communicable diseases, improvement of maternal and child health, development of a district health system, and regulation of the private sector were listed, which may have an impact on newborn survival in the province.

*Process*: Formulation of this policy also considered Millennium Development Goals (MDG) 4 and 5, and its key area 3, “to improve maternal and child health”, only listed the additional role of community midwives to support lady health workers (LHWs).

*Stakeholder/actors*: The policy did not identify the key stakeholders responsible for the implementation of objectives. There was only a mention of inter-sectoral collaboration and community participation to leverage the implementation.

### Sindh Health Sector Strategy 2012–2020

*Context*: The Sindh Health Sector Strategy 2012–2020 [32] is the most recent health policy document produced by the province of Sindh.

*Content*: The strategy promises to offer the minimum and essential health service packages consisting of actions for newborn health such as providing newborn resuscitation services, safe newborn care at birth, newborn check-ups, integrated management of childhood illnesses, BEmONC, early diagnosis of illnesses and stabilization, and referral for CEmONC. There is no identification of the health facilities where advanced newborn healthcare services will be established, even though the document proposes that vouchers would be used to cover the cost of referrals for sick newborns.

*Process*: The policy lacked implementation actions that would have helped establish advanced newborn care units in the province which it promised.

*Stakeholders/actors*: One reason for this deficient link between policy and implementation could be a lack of identification of the responsible and leading institutions, and delegation of the responsibility and authority to steer the implementation process. Because of these weaknesses, the strategy has yet to materialize into effective delivery of newborn healthcare services and impact upon newborn survival indicators.

### Integrated Reproductive, Maternal, Newborn, Child and Adolescent Health and Nutrition Strategy 2016–2020

*Context*: A further relevant policy in Sindh is the Integrated Reproductive, Maternal, Newborn, Child and Adolescent Health and Nutrition Strategy 2016–2020 [33]. This strategy follows the priority areas of National Health Vision 2016–2025, with a broad aim of “providing affordable and quality healthcare services for pregnant women and newborns in accountable and equitable manner through evidence-based operational planning”.

*Content*: The content directly linked with newborn survival in this policy includes upgrading the district and taluka headquarters hospitals to CEmONC or BEmONC facilities, and provision of equipment to health facilities.

*Process*: Despite the promise of establishing infrastructure, there is no item in the policy on newborn care units in any identified districts (or upgrading of existing but nonfunctioning units) or on the mechanism and time frame within which such changes will take place. The major thrust of the policy, as it historically has been, is upon staff refresher training to improve the quality of care by the year 2020.

*Stakeholders/actors*: The policy identified a funding gap of 85.24%, which it proposed to overcome through the use of Sindh government resources and by approaching potential donors for the implementation strategy requiring 117.6 billion Pakistani rupees (702 million US dollars). Considering that the major service providers in Sindh are either the provincial health department, operating most tertiary and secondary hospitals, or the Peoples’ Primary Healthcare Initiative, operating most primary healthcare facilities, and the fact that currently the MNCH programme in Sindh mainly provides midwifery training, it may not be able to drive the establishment and scaling up of newborn care services in the province.

### What is known about successful newborn survival policies in comparable countries?

Considering the lack of progress in implementation of policies for the survival of newborns in Pakistan, we now turn to identifying the policy drivers that have led to effective implementation of newborn mortality preventive actions in comparable countries.

India had an extremely high burden of newborn mortality when the National Rural Health Mission was launched in 2005 with a commitment to reduce neonatal mortality rate by 20 per 1000 live births by 2010 [34]. The Mission instigated improvements in maternal and child mortality in high-focus states through community participatory approaches and reduction in socioeconomic inequities [35]. Under its facility-based newborn care programme, 565 sick newborn care units, 1904 newborn stabilization units, and 14 163 newborn care corners were fully operational by 2015. These units currently provide services such as immediate and lifesaving resuscitation at birth to more advanced care such as mechanical ventilation for babies with complications like sepsis and preterm birth [36]. India has expanded this specialized care network, and currently 793 sick newborn care units treat approximately 1 million sick newborns every year [37]. Special newborn care units were expanded to 214 of the 228 districts in the six states of India with the support of UNICEF. The overall facility-based newborn care programme was allocated 3.162 billion Indian rupees (41.8 million US dollars) by the central government; however, the states could only spend about 63% of this budget because the newborn care units were already established by the state government [38]. The provision of these services contributed to the reduction of newborn deaths by 33% between years 2000 to 2013 [39], and the most recent data show that the neonatal mortality rate in India dropped from 33 in 2010 to 24 per 1000 live births [40].

Newborn care was included as an integral part of the National Rural Health Mission which is accompanied by an implementation framework for the child health strategy in India [41], explicitly outlining areas for integration of newborn care within primary healthcare, as well as resource allocation for activities, and monitoring and accountability. India incorporated the findings of *The Lancet* Every Newborn Series and developed its Newborn Action Plan in 2014 [42], leading to the integration of key interventions within the existing programmes. These interventions included antenatal steroids for preterm births, delayed cord clamping, kangaroo mother care, family participatory care, establishment of lactation management centres, and several other maternal and child health quality improvement initiatives [37]. Our review of policies from Pakistan shows that most of them did not contain such detail as was observed in India’s case. The policy documents from Pakistan also lacked in the use of evidence from other countries, or they did not consider international targets. Bangladesh is another country that could be compared with Pakistan’s journey towards improving newborn survival. Bangladesh reduced its newborn mortality rate to under 22 per 1000 live births over the last decade. The main guiding policy has been the “National Neonatal Health Strategy and Guidelines for Bangladesh”. Released in 2009, it set a neonatal mortality rate target of less than 22 by 2015 [43]. The process of developing the strategy included subnational and national working groups; therefore, the stakeholders owned the implementation process [43]. The strategy document is comprehensive and not only includes the clinical case management guidelines for newborn conditions, but it also provides detailed guidelines for the facility-based supervision, monitoring, and evaluation of newborn healthcare services [44]. The strategy also explicitly listed neonatal mortality rate, neonatal mortality as a proportion of infant and under-5 mortality, proportional cause of neonatal mortality, and low birth weight rate as the four most important impact indicators for reporting. Development of the National Neonatal Health Strategy and Guidelines led to the establishment of at least 32 special care newborn units and newborn units that provide highly specialized care to sick newborns in 25 low-performing districts of Bangladesh. These special care newborn units have played a pivotal role in reducing newborn mortality by providing high-quality specialized care for small and sick newborns referred from lower-level healthcare facilities or directly admitted from the communities [45]. In Pakistan’s case, the specialized care newborn services are available in regional tertiary care hospitals, and patients are referred to these centres from surrounding districts where such services are absent. The establishment of a high-quality newborn care model, with a functioning specialized care service located within districts and connected with lower-level health facilities, has yet to be adopted in Pakistan [22].

## Discussion

Our analysis of key policy documents (Table 1) on Pakistan’s health and maternal and child healthcare in the past 20 years has revealed an absence or a lack of clear and specific goals, targets, plans, and implementation strategies to address the burden of newborn mortality. Some of these goals may not have been realized, especially in terms of implementation of interventions and improvement in service delivery, because they were overambitious or defined without clarity as to what institutions and persons will achieve them and in what time frames. The private sector, international donors, or provincial health departments are most often loosely identified without clarifying the scope of the work and responsibilities. The lack of a monitoring and evaluation mechanism and unclear verifiable goals was another deficiency that we identified in our analysis. Incomplete process of decentralization of maternal and child healthcare service delivery could be one of the most important reasons why the policies drawn after 2010 have not yet impacted newborn mortality in the provinces.

**Table 1:** Summary of analysis of policies and plans with an impact on newborn mortality in Pakistan

Health policy analysed	Context	Content	Process	Stakeholders/actors
National Health Policy 2001	First significant health policy in the decade	Ten strategies including a target to reduce newborn mortality from 25 to 15% by 2010	Broad and nonspecific process of implementation Identified service delivery improvement as key area of intervention	Provincial governments Recruitment of community health workers Ambitious goal to regulate private sector
MNCH PC-1	Major maternal and child health-related policy aligned with MDGs 4 and 5	Aimed to improve community access to healthcare Acted merely as policy guidance Did not influence service delivery	Aims too ambitious for the provinces to implement Ambitious goal to establish EmONC facilities was not fully materialized Lower level of healthcare system did not fully benefit	International donor support for implementation Relied on provinces and donors to support, yet driven by the federal office
National Health Policy 2009	Generic policy without specific implementation strategy	Lacked in items on funding, timeline, or a direct relevance to newborn survival	Although it reiterated the importance of EmONC, it only superficially touched how would it be done	Federal Ministry of Health was abolished after decentralization in 2010
Every Newborn Action Plan (WHO/UNICEF)	Key guiding policy for countries signing to it implement strategy	Strategic objectives comprehensively address all aspects of newborn survival including reducing inequities	Pakistan’s progress on the plan was dismal Major flaw like in other policy implementation has been the time frame	Endorsement by 194 Member States including Pakistan
National Health Vision Pakistan 2016–2025	A federal policy developed recognizing a lack of progress in the provinces after decentralization	Newborn healthcare services an important outcome, yet no clear service level target	A lack of identification of monitoring and evaluation of implementation Policy seemed to have gathered a little momentum	Vague listing of wide-ranging private sector involvement
Miscellaneous health-related policies with a possible impact on newborn health	Various policies with potentially an indirect impact upon newborn survival	Conspicuously lacking newborn survival as the key outcome	Detailed implementation and evaluation process generally missing	UN and international donors developing documents with little commitment from the key public sector stakeholders
Provincial MNCH Programme after decentralization	Decentralization of programme, with inadequate role definitions and resource allocation	Progressive limitation of the programme mandate to only teaching and training of midwives	Financial constraints and difficulties post-decentralization led to these limitations to the scope of work	Declining support by the donors that financed the federal programme previously
The Sindh Health Policy 2005	First provincial health policy linking child health with MDGs	Despite considering child health a priority, newborn health was missing in the document	Vague implementation mechanisms Vague reference to improving child health without pinpointing the key areas of service delivery	Mention of inter-sectoral collaboration, yet no clear roles and responsibilities identified
Sindh Health Sector Strategy 2012–2020	Most recent provincial strategy	Addresses the need to improve service delivery by introducing health service packages, yet key information about establishment of EmoNC centres is missing	Implementation actions are largely missing	A failure to identify clear roles and responsibilities Monitoring and evaluation mechanism unclear
Integrated Reproductive, Maternal, Newborn, Child and Adolescent Health and Nutrition Strategy 2016–2020	The first policy that addresses the key issues of access to and quality of MNCH services	Clearer aims to upgrade health facilities, yet no commitment to establish specialized care units for sick newborns	Time frames not available Refresher trainings one of the major strategies Concrete and objectively verifiable goals not identified	Looks to donors for filling the resource gaps

Our analysis highlights that Pakistan's health policies failed to include time-bound targets for the reduction of newborn mortality following Pakistan’s endorsement of global newborn survival goals and targets. Successful policies in comparable countries were more elaborate and supported with subsequent development of implementation frameworks with greater ownership of the policy implementation process. Our assessment of the health policies in Pakistan indicates confusion about roles and responsibilities of institutions in the implementation process and accountability for the outcomes. A partial decentralization of healthcare services, including maternal and child healthcare policy-making and service delivery, has exacerbated this problem. There have been some instances of reversal of the decentralization process; and in other instances, newly decentralized institutions, like the MNCH programme, struggled to find adequate support from provincial ministries of health. This piecemeal decentralization, often coupled with limited sharing of authority, resources, and decision-making, leads to challenges of inadequate policy-making and poor health governance, impairing service delivery [46]. Good health governance in turn is essential to steer newborn survival in settings with a high newborn mortality burden [47]. Poor governance impairs the health system and the ability of the decision-makers to develop and implement key policies for newborn survival [1]. Clarity around the decentralization process with corresponding devolution of authority and resources, as well as responsibilities, is required, and provinces need to reorient their newborn survival policies according to the burden of newborn mortality and morbidity, local culture, and healthcare system context.

Even though the Pakistan government produced policy documents at regular intervals, which focussed upon saving the lives of mothers and children around birth, these documents conspicuously lacked an explicit strategy to deal with an unacceptably high neonatal mortality. It is also pertinent to note that major national health policies as well as health vision documents give inadequate insight into what intervention packages would be implemented, with what resources, and by which institutions [12]. Prior to decentralization, the MNCH programme appeared functional at a federal level, but the transition to a decentralized system exposed a lack of policy details, implementation strategies, and integration with the local health services in the districts [24]. This is evident in a post-decentralization scenario in which the MNCH programme has cut most of its service delivery responsibility in the provinces.

One of the reasons that the health policies in Pakistan were not systemically drafted and had wide gaps in the areas of target-setting, implementation mechanism, and evaluation was that the policy-making failed to fully realize international commitments to newborn survival. This could be because policies have been traditionally shaped by the political culture in Pakistan rather than by following evidence [12]. Our analysis of newborn survival policies impacting upon the improvement of newborn care services and newborn indicators in India and Bangladesh shows they were improved because of the international commitment driving a successful implementation process. Both India’s and Bangladesh’s policies on newborn interventions were influenced by international strategies like the Newborn Action Plan and *The Lancet* Every Newborn Series. Pakistan’s endorsement of international newborn survival commitments obliges the country to adopt the commitments and guide future policy formulation. Another factor that was common to India’s and Bangladesh’s progress on implementation and scaling up of newborn care services was comprehensive and practical policy-making, and ownership of the policies by the main stakeholders in the implementation process and valuing the local context. Although agencies like UNICEF have supported facility-based newborn care in both countries through technical resources and some equipment, one of the major differences in programme implementation was the adequate budgetary management by their governments. This comparison supports the fact that similar progress is possible in Pakistan.

## Strengths and weaknesses

A lack of involvement of stakeholders, including the policy-makers, politicians, and health sector leaders, might have strengthened the methodological approach and findings of this study, but such an approach was not possible when this analysis was conducted. Another weakness of this analysis was that we may have missed some of the strategic documents which were not available online or are in development. Using the main health policy documents spanning a period of the past two decades, considering the decentralization context to evaluate these policies, and comparison of achievements in newborn survival with those of the regional countries are some of the strengths of this analysis. However, we could only access documents available online or received with personal requests to the relevant departments. Also, the wider impact of the larger factors including political factors and social determinants of health such as education, nutrition, or related policies and programmes was not evaluated, as they were not within the scope of this study; however, they have a great impact upon the success of any policy.

## Conclusion

In conclusion, health policies and strategies of Pakistan, including those of Sindh province, lack the necessary clarity and direction for the implementation of the available evidence-based interventions to prevent a persistently high burden of newborn illness and mortality. In comparable countries too, the policies were impactful because they identified achievable and time-bound targets for newborn mortality rate and then explicitly listed the interventions that would be integrated within existing public healthcare system. Clarity surrounding decentralization, including appropriate institutional responsibility and accountability, and implementation of commitment to international newborn mortality prevention targets can guide Pakistan’s health policies to save newborn lives in the future. This research may guide maternal and child health-related policy formulation in Pakistan and other similar settings with a high burden of newborn mortality. Future policies on newborn survival in Pakistan may not be impactful without including key interventions providing detailed frameworks for implementation at lower healthcare system levels. The desired goals should be transformed into implementation plans and strategies, including specific programmes, interventions, and clinical standards, and separate clinical guidelines for health workers.

## Data Availability

No datasets were used and/or analysed for this review.

## Abbreviations

BEmONC: Basic emergency obstetric and newborn care
CEmONC: Comprehensive emergency obstetric and newborn care
DFID-UK: Department for International Development–United Kingdom
LHW: Lady health worker
MNCH: Maternal, newborn, and child health
UNICEF: United Nations International Children's Emergency Fund
